# Thoughts on the Study of Diseases, with Reference to Geology

**Published:** 1852-01

**Authors:** J. B. Hiester


*Thoughts on the Study of Diseases with reference to Geology. Communicated to the Medical Society of Berks County, Nov. 4th,* 1851, *by* J. B. Hiester, M. D.

It is assumed as an established fact in Geology that soil is a detritus, the result of disintegration of the solid strata that compose the crust of the earth. With scarcely an appreciable exception, (beyond the drift region) the soil thus formed rests immediately upon the rocks from which it has been produced. That soil owes its variety to the varied constituent parts of which the underlying rocks are composed, was very reasonably inferred from its position and physical character. Chemical analysis, on a very extended scale, has fully sustained the truth of this influence. It is also proved by analysis, that all foreign matters or impurities, found in water, have their source in the soluble materials contained in the geological formations through which it percolates. An examination of the soil or water of any district readily determines its geological constitution; so also does a knowledge of its geology lead to certain conclusions as to the character of its soil and water. Any power, therefore, attributable to the action of soil and water, has its source in geological formation.

The well-marked influence exerted by soil and water upon the vegetable kingdom, is so obvious, that it must have obtruded itself upon the observation of man, from the earliest period of his history. That the limits of plants are distinctly and sharply marked by this influence is well known to every botanist. This limitation is not restricted to herbaceous plants only, but is clearly visible also in the distribution of the forest-trees, many of which are almost exclusively confined to certain soils. In the aquatic plants too, the same limitation is clearly marked. In some streams we find certain species of plants, which are scarcely ever met with in other streams, in their immediate vicinity. All the physical conditions of such streams are often precisely similar, differing only in the chemical character of their waters.

From time immemorial has the agriculturist been guided in his occupation by a knowledge of the fact, that certain kinds of natural productions require a peculiar soil for their successful cultivation. An attempt to cultivate particular species of the cerealia, for example, in some soils, is certain to result in failure, whilst other species produce abundantly when sown upon the same soil. These facts were so well known from observation, that they were unhesitatingly and advantageously acted upon. But the science of chemistry has not only confirmed their truth, but clearly revealed the laws upon which they depend.

Many of the cerealia, as well as many roots and vegetables cultivated for food, indispensibly require certain inorganic chemical constituents to build up their organization. These peculiar constituents are derived from the soil. Those soils that are destitute of the principles demanded by the particular plant to be cultivated, are sterile as regards that plant, and yet may be perfectly congenial to, and highly fertile for other plants, demanding other elements for their growth and perfection. Upon these well established facts rests the whole beautiful modern science of agriculture.

If then, geological formations exert an influence so clearly and distinctly marked upon the vegetable world, we should naturally look for a similar power exerted upon the animal kingdom.

In the lower orders of the animal creation, this influence is very obvious. Zoologists have long since well ascertained, that the distribution of testaceous mollusks, both terrestrial and fluviatile, is clearly traceable to the power exercised by geological formations. These animals may be scarcely said to have an existence in granitic districts, whereas in calcareous formations, they are exceedingly abundant. In the case of the fluviatile genera, as in aquatic plants, peculiar species are found in particular streams. It is a well known fact also, that certain species of fish inhabit certain streams, and are seldom or never detected in contiguous streams, although their waters are discharged into the same rivers or lakes. Even in the same lake, certain species occupy peculiar localities, in which other species are scarcely ever seen. It appears to me that the cause of these curious restrictions may be legitimately referred to a peculiar constitution of the waters.

How far the higher orders of animals are operated upon directly by geological constitution, has not yet been ascertained, for want of due observation, but I am strongly inclined to think that proper attention directed to the subject would develop highly interesting and important facts. The indirect influence exerted through the medium of the vegetable kingdom, the source of sustenance of most animals, cannot be doubted.

It is well known that the animal organization, like that of plants, absolutely requires a large amount of certain inorganic substances for its development and well-being, derived, partly from the soil, indirectly through the medium of the vegetable kingdom, and partly directly from the water. We also know that the substances thus demanded are among the most decided physiological agents, as phosphorus, chlorine, fluorine, sulphur, soda, potash, lime, iron, &c. Another fact familiar to every practitioner is, that a deficiency in the human system of at least some of the above named substances, induces pathological conditions strongly marked by distinct pathognomonic signs; and that these signs are truthful indices, is proved by the fact that a supply of the deficient substances reproduces a normal condition of the system.

If, then, a deficit of these inorganic matters, and those among the least active, produces well-marked pathological conditions, it is very conceivable that an undue accumulation in the system of those much more active, should produce even a more decided effect. That such accumulations do occur, is more easily supposed, than the established fact that deficiencies occur. A long and uninterrupted residence upon certain geological formations, where the inorganic substances required by the system are either superabundant, very deficient, or even wanting, must necessarily produce a surcharge or deficit of them, and consequently are abnormal states.

By the light of recent discovery it appears that nature does not confine herself to a general distribution of those substances only, which are known to be imperiously required for the healthy existence of the animal organization, but that other powerful agents exist in a form to be readily received into its composition. Thus iodine, a very efficient agent, has been detected in various species of inland aquatic plants, among which is one at least, the nasturtium officinale, that is largely eaten as a salad. It is not at all improbable too, that bromine, so nearly allied to iodine and fluorine, will be found to be more generally distributed than was formerly supposed.

Here it appears to me is opened a wide and promising field for accurate observation, and above all, for the exercise of the late improved methods of quantitative chemistry. A close and careful investigation of the subjects hinted at in the foregoing remarks, holds out to my mind very flattering prospects of arriving at certainty in regard to the causes, cure and prevention of endemic and epidemic diseases, so long enshrouded in mystery and obscurity.

The hasty and necessarily imperfect observations collected by your Sanitary Committee of last year, of which I had the honor to be chairman, have already produced some encouraging results by connecting the study of diseases with geology. The well defined restriction of certain endemic and epidemic diseases to certain geological formations of our county, the almost entire exemption from some common affections on others, the remarkable difference of average longevity on different strata, are well calculated strongly to arrest the attention of the medical inquirer.

Should the conjecture thrown out as to the manner in which the system is effected by different strata, the source of soils so very different in their chemical composition, and of waters holding in solution the most opposite substances, prove fallacious, yet the facts just stated are sufficiently prominent to incite to more precise and closer investigation.

In all scientific researches an important step is to contract the compass of the field of investigation, and clearly to define its boundaries. The vagueness in our investigations of endemics and epidemics has too often resulted from the general and indefinite manner in which they have been conducted. The study of diseases, as they are confined to, or modified by geological formations, would so circumscribe the field of investigation as probably to lead to more definite conclusions.

I cannot resist on this occasion again to express my strong commendation of the resolution offered by Dr. Isaac Parrish to the State Medical Society at their last session, and adopted by that body, recommending the County Societies to procure geological surveys of their respective counties to serve as bases of their topographies. All topographies that are not founded on geology, are certainly and manifestly defective. Could we procure topographical histories upon this basis of every county in the State, we should have a beautifully connected and intelligible system of great interest, even independently of the pathological results that may so resonably be expected.

